# The International Association for the Study of Lung Cancer Early Lung Imaging Confederation

**DOI:** 10.1200/CCI.19.00099

**Published:** 2020-02-06

**Authors:** James L. Mulshine, Ricardo S. Avila, Ed Conley, Anand Devaraj, Laurie Fenton Ambrose, Tanya Flanagan, Claudia I. Henschke, Fred R. Hirsch, Robert Janz, Ryutaro Kakinuma, Stephen Lam, Annette McWilliams, Peter M.A. Van Ooijen, Matthijs Oudkerk, Ugo Pastorino, Anthony Reeves, Patrick Rogalla, Heidi Schmidt, Daniel C. Sullivan, Haije H.J. Wind, Ning Wu, Murry Wynes, Xie Xueqian, David F. Yankelevitz, John K. Field

**Affiliations:** ^1^ Rush University, Chicago, IL; ^2^ Accumetra, LLC, Clifton Park, NY; ^3^ University of Liverpool, Liverpool, United Kingdom; ^4^ University College, London, United Kingdom; ^5^ GO2 Foundation, Washington, DC; ^6^ Canadian Partnership Against Cancer, Toronto, Ontario, Canada; ^7^ Icahn School of Medicine at Mount Sinai, New York, NY; ^8^ Mount Sinai Health System, New York, NY; ^9^ University of Groningen, Groningen, Netherlands; ^10^ National Cancer Center Hospital, Tokyo, Japan; ^11^ University of British Columbia, Vancouver, British Columbia, Canada; ^12^ Fiona Stanley Hospital, Western Australia; ^13^ University Medical College, Groningen, Netherlands; ^14^ Instituti Tumori, Milan, Italy; ^15^ Cornell University, Ithaca, New York, NY; ^16^ Toronto Joint Department of Medical Imaging, University of Toronto, Ontario, Canada; ^17^ Duke University Medical Center, Durham, NC; ^18^ National Cancer Center, Peking Union Medical College, Beijing, China; ^19^ International Association for the Study of Lung Cancer, Denver, CO; ^20^ Shanghai General Hospital, Shanghai, China

## Abstract

**PURPOSE:**

To improve outcomes for lung cancer through low-dose computed tomography (LDCT) early lung cancer detection. The International Association for the Study of Lung Cancer is developing the Early Lung Imaging Confederation (ELIC) to serve as an open-source, international, universally accessible environment to analyze large collections of quality-controlled LDCT images and associated biomedical data for research and routine screening care.

**METHODS:**

ELIC is an international confederation that allows access to efficiently analyze large numbers of high-quality computed tomography (CT) images with associated de-identified clinical information without moving primary imaging/clinical or imaging data from its local or regional site of origin. Rather, ELIC uses a cloud-based infrastructure to distribute analysis tools to the local site of the stored imaging and clinical data, thereby allowing for research and quality studies to proceed in a vendor-neutral, collaborative environment. ELIC’s hub-and-spoke architecture will be deployed to permit analysis of CT images and associated data in a secure environment, without any requirement to reveal the data itself (ie, privacy protecting). Identifiable data remain under local control, so the resulting environment complies with national regulations and mitigates against privacy or data disclosure risk.

**RESULTS:**

The goal of pilot experiments is to connect image collections of LDCT scans that can be accurately analyzed in a fashion to support a global network using methodologies that can be readily scaled to accrued databases of sufficient size to develop and validate robust quantitative imaging tools.

**CONCLUSION:**

This initiative can rapidly accelerate improvements to the multidisciplinary management of early, curable lung cancer and other major thoracic diseases (eg, coronary artery disease and chronic obstructive pulmonary disease) visualized on a screening LDCT scan. The addition of a facile, quantitative CT scanner image quality conformance process is a unique step toward improving the reliability of clinical decision support with CT screening worldwide.

## INTRODUCTION

Lung cancer is the most lethal cancer throughout the world, and it typically presents at a late stage when cure is unlikely.^1,2^ Recent reports have demonstrated the usefulness of low-dose computed tomography (CT) screening in reducing lung cancer mortality in heavily tobacco-exposed individuals.^3-7^ However, to achieve the most efficient screening management, groups have incorporated a quantitative assessment of pulmonary nodule volume to guide the diagnostic case–finding efforts within the screening process.^8-12^ In this fashion, the false-positive detection rate can be reduced from 28% to approximately 3%.^10-12^ This improves the screening cost and reduces the potential for iatrogenic harm, which would be a critical aspect to include in the global implementation of computed tomography (CT) screening.^13^ The concept of developing an early lung cancer image registry emerged through a series of workshops sponsored by the International Association for the Study of Lung Cancer (IASLC) as a critical opportunity to accelerate the pace of innovation in improving the curative management for detection and intervention with early lung cancer.

The IASLC mission is to improve lung cancer outcomes through international and multidisciplinary collaborative efforts. This large collaborative image archival and analysis effort builds on the established IASLC successes with the national and international lung cancer IASLC TNM Staging Committee, which has been undertaken in collaboration with the Union for International Cancer Control and American Joint Committee on Cancer, as well as with the recently updated lung cancer pathology collaboration with the WHO.^14,15^ Success in developing screening tools, as with the staging and pathology efforts, involves issues of scale and cost that leverage the IASLC’s broad international scope and expertise in aligning global participation to improve early lung cancer management.

**Context**

**Key Objective**
Can an open-source cloud-based environment become a repository of screening computed tomography images and associated data to enable quantitative and related imaging tool development for use in guiding the management of early, presymptomatic lung cancer and related thoracic diseases?
**Knowledge Generated**
A prototype international, open-source imaging resource is proposed that can allow federated image/data interrogation. This construct is designed to comply with existing international standards for data security while enabling the development and validation of new imaging biomarkers to facilitate early lung cancer management.
**Relevance**
Lung cancer screening is emerging as an important approach for early lung cancer management. However, robust and economic image analysis tools are needed to ensure facile clinical workflows for pulmonary nodule detection and quantitative nodule assessment so that this new service can be provided to the target high-risk population at high quality throughout the world.

To date, no consensus image analysis tool has emerged to allow routine and reliable volumetric characterization of pulmonary nodules in routine clinical imaging settings. Developing a tool to allow easy and robust nodule measurement requires access to large numbers of high-quality thoracic CT images that were acquired with the intent of precisely measuring volumes of pulmonary nodules 5 to 10 mm in diameter. Because this is a newly appreciated opportunity as lung imaging technology rapidly improves, lung images from earlier CT screening trials were generally not acquired with sufficient resolution to enable this precise volumetric tool development. Therefore, prospective collections of CT images from current-generation, high-resolution CT scanners are urgently required to address this gap. This lack of large quantities of such high-quality image data imposes a profound barrier to progress with early lung cancer management.

### How to Bring Value to Early Lung Cancer Detection

In response to this situation, the IASLC hosted a planning workshop held in Dallas, Texas, in February 2018. Although aware of the heterogeneous nature of existing image registries at leading centers from around the world, the group proposed the creation of a cloud-based informatics infrastructure to interact with existing international registries and centers collecting thoracic CT images together with associated core clinical outcomes data to optimize cost and data security.

This IASLC Early Lung Imaging Confederation (ELIC) was proposed as a hub-and-spoke architecture with the intention of enabling the imaging-donating local site to retain all of the images and metadata within their defined spoke environment consistent with local governing data-sharing provisions. With local site permission, their stored clinical and imaging data can be made accessible, to allow software tools distributed to the spoke from the hub to the relevant stored digital data. In this confederated architecture, the hub acts as a conduit with the spokes to distribute software analysis tools to the relevant spokes and then to aggregate the results of the analysis of the images stored locally in the participating spokes. Therefore, only the resulting analysis data will leave an individual spoke. The resulting analysis data aggregated in the hub from multiple sites with diverse populations allow for research and for quality questions to be addressed with a potentially vast number of test screening cases from multiple countries.

To accommodate this architecture, the most cost-feasible approach for a flexible, scalable, and sustainable environment capable of enabling the goals of ELIC is to leverage the global accessibility of a cloud environment.^16^ Currently, thoracic CT screening images and associated clinical outcomes and relevant metadata are stored in a vast array of architectures across IASLC member sites. We envision developing a vendor-neutral, secure, scalable, cloud-based environment to bridge to existing sites’ data storage resources. Table 1 summarizes the design considerations guiding the development of this informatics resource. Given the dynamic and complex nature of the privacy challenges inherent in collecting and sharing large amounts of imaging and clinical data, the proposed IASLC imaging/data resource may be preferable for many national sponsors to have a rigorously designed, precompetitive environment hosted by an international, nonprofit professional society such as IASLC. IASLC has a proven legacy of patient benefit and as a reliable host to ensure appropriate stewardship as an “honest broker” for such a critical international resource.^14,15^

**TABLE 1.**
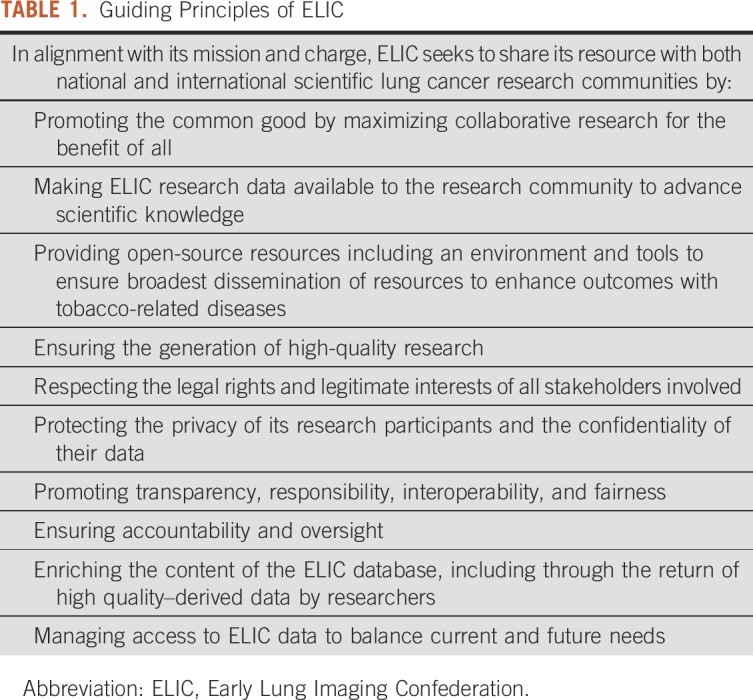
Guiding Principles of ELIC

### High-Quality Image Acquisition

For optimal efficiency, there is also the need to prospectively ensure that newly accrued thoracic CT images are objectively of sufficiently high quality to support robust quantitative analysis. Because ELIC is envisioned to be a resource to develop and then validate image processing algorithms as required to support clinical decision making for early lung cancer management, such efforts will move into the rapidly evolving realm of deep learning methods or artificial intelligence approaches; therefore, the curation of an image collection that has been optimized for quantitative measurement is critical.^17,18^

Reliable guidance and clinical management in the lung cancer screening setting requires accurate and robust analysis of pulmonary nodule volume. Therefore, ELIC will require image-sharing sites to prospectively adhere to the Quantitative Imaging Biomarkers Alliance (QIBA) of the Radiologic Society of North America (RSNA) CT Small Lung Nodule Profile quality provisions to ensure that aggregated imaging data obtained for quantitative analysis are of a high quality; this is a unique strength of the ELIC effort.^19,20^

From extensive work in optimizing image measurement quality in this setting, the QIBA has developed a process to address these factors.^17,18^ This entails analyzing pulmonary nodules in the volume range relevant to lung cancer screening (6-10 mm in diameter). Crowd-sourced data collected from international QIBA CT Small Lung Nodule testing sites have been used over the past 2 years to help identify and globally disseminate the top-performing CT image acquisition protocols for each CT scanner model. This innovative approach has enabled what we believe to be the first rapid global optimization of scanner image acquisition protocols for CT lung nodule measurement. The data are also revealing new insights into low-dose CT lung cancer screening image acquisition performance, including the reproducibility of image acquisition properties, differences between requested and obtained slice thickness, and losses of resolution associated with lowering the radiation dose.^21^

## METHODS

**FIG 1.**
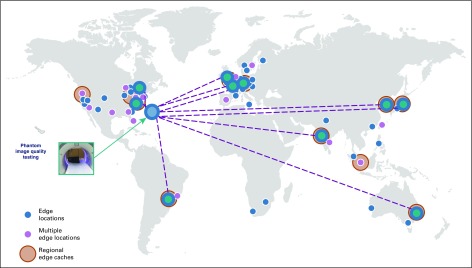
The global distribution of the hub and spokes for the 2018 World Conference on Lung Cancer ELIC proof-of-concept demonstrations using the Amazon Web Services (AWS) cloud.

A proof-of-concept demonstration was developed in 2018 to evaluate the potential of a hub-and-spoke–distributed lung cancer screening image archive and computing architecture to achieve the goals of ELIC^19^. Ten international cloud computing sites were chosen from the Amazon Web Services global cloud network map to launch and set-up spoke EC2 cloud computing instances, as shown in Figure 1. This figure overlays the 10 ELIC spokes feeding a central hub (at the point of the arrow) on the Amazon Web Services (AWS) global cloud network map. The hub is shown (with the blue circle at the tip of the arrow), with each of the 10 spokes (indicated by green circles) populated with an identical set of 100 publicly available de-identified CT lung scans. As shown on the map, existing AWS cloud services sites are indicated by smaller (lighter) blue and purple circles. This distribution of existing cloud resources demonstrate the global reach of AWS to support local/regional hosting of available lung cancer screening images and corresponding clinical data as required by General Data Protection Regulations. Each of these spokes was populated with an identical set of 100 publicly available de-identified CT lung scans.^20^ However, each scan was given a unique patient ID and fictitious age, sex, and pack-year demographics. This was done so that proof-of-concept performance testing could be obtained with the equivalent of 1,000 patient cases; the analyses described in this report were repeated on at least 5 separate occasions. In addition, a Hub EC2 cloud computing instance was set up at the Northern Virginia location of the Amazon Web Services cloud.

**FIG 2.**
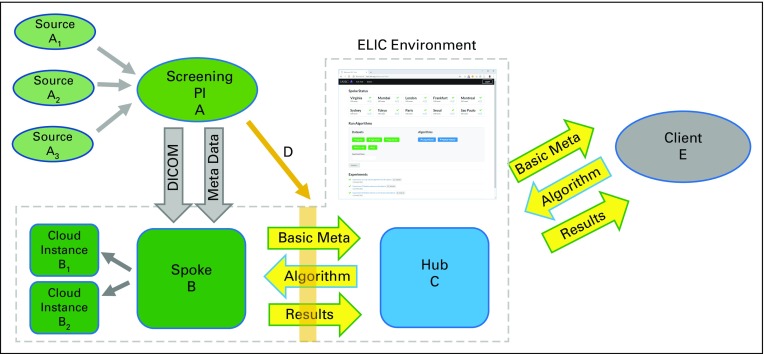
High-level illustration of the ELIC Hub & Spoke Environment and how lung cancer screening principal investigators (PI) and clients will interact with the system. DICOM, Digital Imaging and Communications in Medicine; ELIC, Early Lung Imaging Confederation.

Figure 2 is a schematic representation of the computing resources and main data flows that were used during the design of the ELIC Hub-and-Spoke Environment (H&SE) software. A lung cancer screening principal investigator (PI; A) typically coordinates CT lung cancer screening for several screening sites shown as sources (Ai). The PI is responsible for submitting de-identified lung cancer screening image DICOM (Digital Imaging and Communications in Medicine) data and metadata, including any requested image annotations, before data upload to a spoke (B). Each time data on the spoke (B) are added or corrected, a set of basic demographics and metadata information about the new data is communicated to the hub (C). In this way, the hub and spoke will remain in agreement regarding the data that a PI has made available for analysis on the spoke (B). Because the spoke (B) can be set up on a local cloud computing instance or on local computing hardware, the spoke (B) data will remain within the source geographic region and will only be analyzed according to strictly administered analysis and reporting rules (D) decided by the PI (A). The data residing on all spokes are de-identified by the PI (A) before upload.

Thus, at all times, only de-identified data are stored on the ELIC H&SE and, at rest, the data are also encrypted. Each ELIC spoke contains a de-identified set of data stored on a spoke using identical ELIC H&SE data storage organization and methods. This allows the ELIC H&SE software to contain fully automated scripts for thoroughly performing required data protection operations, such as the General Data Protection Regulation “right to be forgotten.” Each ELIC spoke will have automated scripts that achieve the data governance protections and procedures required by local regulations, some of which will be standard and can be provided by the same ELIC H&SE script for all regions.

A client (E) can view a listing or catalog of all the data sets available within the ELIC environment and take the necessary steps to run a quantitative algorithm on all the data or on a subset of the data available within the ELIC environment. This is referred to as running an “experiment” within the ELIC environment. To run an experiment, the client (E) must provide a quantitative algorithm according to ELIC H&SE specifications to the hub (C) and request that the hub execute the experiment on all the relevant spokes (B). The hub (C) then coordinates the running of the quantitative algorithm on the spokes and collects the results according to the analysis rules (D) agreed to by the PI. When the experiment is finished, the client (E) is provided an aggregate summary of all the data collected from the experiment, including information on any errors or issues encountered while running the experiment. A prototype hub password-protected Website^22^ was created that displays the status of spokes, allows the specification and launching of quantitative lung imaging experiments on global collections of data, and provides a real-time quantitative and graphic display of the results obtained from the international spoke calculations.

Two cloud-based experiments using software algorithms were created to accept DICOM data and produce quantitative results and images in a standard format that the ELIC H&SE supports. One algorithm quantitatively measured total lung volume, and the second algorithm quantitatively measured the volume of solid pulmonary nodules. This permitted the ELIC H&SE to demonstrate running the quantitative imaging experiments on image collections at globally distributed spokes and then aggregating the quantitative CT image measurements and output image results on the hub. Each of the 10 spokes was set up to run one of the 2 quantitative CT lung image measurement algorithms when requested by the hub.

The project also deployed the QIBA CT Small Lung Nodule Profile Conformance Certification service on the hub and performed CT image quality conformance assessment calculations on CT phantom scans. Cloud-based phantom analysis software was developed to perform a low-cost CT image quality assessment using a specifically designed phantom (ie, test object). This approach makes achieving CT image quality conformance with the QIBA Small Lung Nodule Profile possible from virtually any clinical imaging site in the world.^17,18^ The purpose of this quality control step is to ensure consistent image quality appropriate for the defined context of use with lung cancer screening–related quantitative assessment for imaging sites around the world. The standardized assessment of CT lung imaging quality is enabled using a dedicated, low-cost phantom (CTLX1) that was developed for this purpose. The CTLX1 phantom contains small, precision-made, geometric components to assess thoracic CT imaging performance. A CT scan of the CTLX1 phantom is typically acquired in approximately 5 minutes and then uploaded to the QIBA Phantom Analysis Service, which rapidly analyzes the uploaded phantom image using automated software to characterize the quality of a CT scanner and acquisition process. The results of the comprehensive QIBA CT image quality analysis are sent back to the site within a few minutes using an easy-to-interpret structured report indicating whether the CT scanner and acquisition parameters were of sufficient quality for quantitative assessment. If the image quality was insufficient, then remediation measures are suggested to the site to improve image quality. This analysis has been performed at > 60 CT lung cancer screening sites evaluating the use of the CTLX1 phantom, including screening sites in Australia, England, Canada, China, Israel, Italy, the Netherlands, Poland, Spain, Japan, and the United States. The image quality data collected from the analysis of CTLX1 phantom scans include CT scanner image acquisition parameter settings such as milliampere, peak kilovoltage, slice thickness, slice spacing, and reconstruction kernel, as well as fundamental image quality characteristics achieved, including levels of edge enhancement, 3-dimensional (3D) resolution, 3D resolution aspect ratio, CT linearity, noise, and 3D spatial warping. Each of these fundamental image quality properties is measured throughout the CT scanner field of view at 3 distances from isocenter (0, 100, and 200 mm) to ensure that lung nodules present in the lung periphery, which is common, can be accurately measured.

For proof-of-concept testing, the QIBA CT Small Lung Nodule Profile Conformance automated phantom analysis software was placed on SPOKE 1 running in northern Virginia and used to run automated image quality analyses on scans of the QIBA CT Small Lung Nodule Profile CTLX1 phantom. These tests confirmed that the fully automated QIBA CT Small Lung Nodule Profile conformance certification methods for CT image quality assessment will be able to successfully run on future ELIC spokes (or hub) running on the Amazon cloud.

A total of 5 live demonstrations were run using the ELIC environment at 5 distinct time windows to evaluate the ability of ELIC H&SE to perform useful quantitative imaging computational experiments on large collections of globally distributed CT lung cancer imaging cases without moving data out of geographic regions. Each demonstration was run for ELIC H&SE analyses performed with a central hub server and 10 globally distributed spoke servers all running on the Amazon Web Services cloud. The prototype ELIC H&SE Website^22^ allowed a user to launch a computational experiment request on a specific set of data distributed across any number of spokes, store the results, and display an aggregated summary report of the results when the analysis was complete.

**FIG 3.**
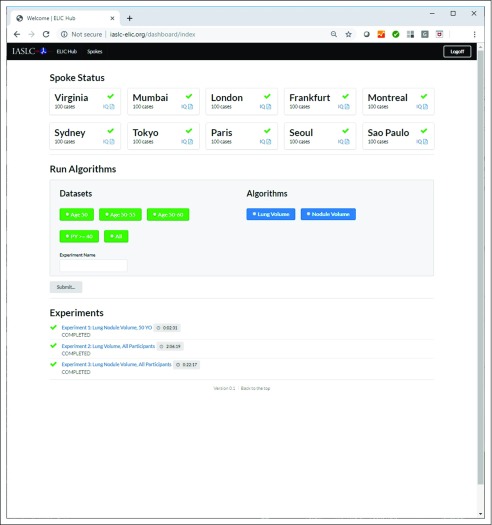
The main user page available at www.iaslc-elic.org for the launch and review of quantitative lung imaging experiments on globally distributed spokes.

In all, 3 pilot testing runs were performed. For these pilot testing runs, quantitative lung volume analyses were completed on 1,000 globally distributed CT lung cancer screening data sets in < 25 minutes at 10 different ELIC hosting sites from 4 continents. Figure 3 shows the main user page available within the Website,^22^ which includes a listing of the available spokes, a place to specify the launch of a quantitative experiment on the data located on the spokes, and a list of pages showing the results obtained from each experiment.

**FIG 4.**
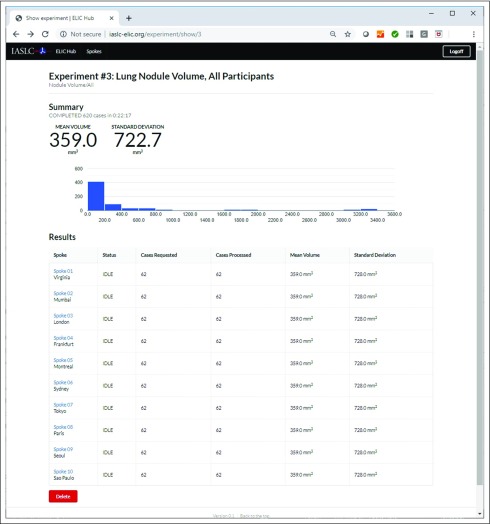
Results page for a lung nodule measurement experiment performed in the ELIC Hub and Spoke Experiment.

**FIG 5.**
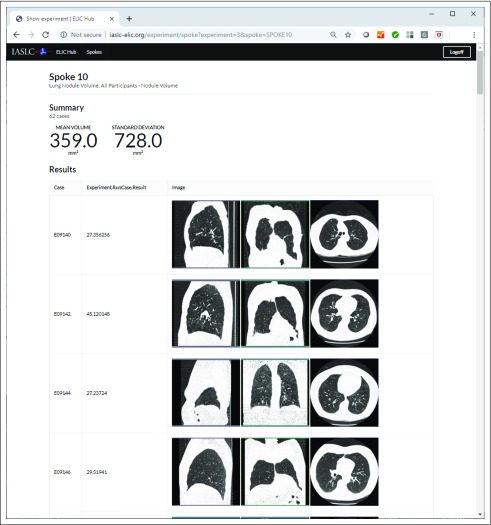
Detailed results for cases run on spoke 10 (Sao Paulo) for experiment 13.

The developed H&SE software allows a hub server to make quantitative CT imaging computational requests to a collection of globally distributed spokes, each of which is populated with de-identified CT lung images. The H&SE software is set up so that each spoke performs a computational request and returns quantitative results back to the hub. Figure 4 shows the results page from a representative lung nodule measurement experiment (experiment 3) performed on all the images at the 10 sites in the archive with lung nodules (N = 62 × 10) in the ELIC H&SE. The results reported were mean volume and standard deviation for all the data sets analyzed. Figure 5 displays the detailed quantitative imaging results that were computed on CT lung imaging cases on SPOKE 10 (Sao Paulo, Brazil) for the same experiment as that shown in Figure 4 (experiment 3), including the quantitative lung nodule volume values and 3 orthogonal reformat images with contours of the segmentation results overlaid for each case.

Three presentations, including a live demonstration, were given at IASLC’s 2018 World Conference on Lung Cancer in Toronto, Canada. In total, > 5 live demonstrations successfully showed the simultaneous running of the CT lung nodule algorithm on CT lung imaging cases distributed at 10 internationally distributed spoke sites.^21^

## DISCUSSION

This initial proof-of-concept project demonstrated the potential of the ELIC H&SE to provide a useful resource for global quantitative lung imaging studies. This project used global cloud-computing resources, each populated with an identical data set of 100 publicly available lung cancer images. A central hub Website simultaneously distributed 2 open-source quantitative lung measurement algorithm requests to the 10 globally distributed spoke sites. Using publicly available CT lung images allowed the project to achieve results quickly. The hub received and aggregated all quantitative algorithm results from the spoke sites and displayed the results on the hub Website. In addition, the QIBA CT Small Lung Nodule Profile CT image quality conformance testing service was successfully run on a spoke to demonstrate that CT image quality monitoring and optimization can be supported with the ELIC H&SE.

Although the developed proof-of-concept software implemented a basic H&SE, the functionality represents a subset of that which will be needed to fully realize the IASLC ELIC H&SE vision. For example, the demonstration Website supports only 1 user type; when mature, the ELIC H&SE will support a variety of different roles and types of access for clients, hub personnel, and spoke personnel. Future versions of the ELIC H&SE will also provide higher levels of security to prevent access or extraction of PI CT image data as well as to prevent unauthorized use of client algorithms.

For these initial demonstrations, the client algorithms were created as open-source projects and built on each spoke into an executable application. However, a future ELIC H&SE will likely also support the distribution of algorithms from the hub. For example, the distribution of virtual containers with executable applications embedded is being explored. Future H&SE environments will also need to provide additional functionality and support for artificial intelligence development and specifically, deep learning algorithm development and testing. A fuller description of the methodology, governance, data use provisions, scope, and other proposed policy elements are provided in the Data Supplement.

The ELIC H&SE prototype vehicle was developed with IASLC seed funding to support feasibility testing and was developed as an open-science and open-source research/care environment to allow a broad range of collaborative participation in achieving its CT imaging-related objectives. Funding to sustain this large-scale international collaborative effort will be dependent on the early lung cancer research and innovation communities using ELIC in their research to address important research and lung cancer care issues. We expect many stakeholders, including software developers, artificial intelligence companies, imaging researchers, diagnostic device developers, medical imaging companies, government agencies, pharmaceutical developers, academic societies, and many other innovators, to be early users of the flexible capabilities of ELIC. This service model is designed to allow a sustainable path forward for this internationally accessible quantitative imaging environment to emerge as a core resource for improving the detection and management of early thoracic disease.

The internationally federated ELIC hub-and-spoke system has features such as robust data privacy provisions to permit large-scale analyses of clinical CT images with relevant associated data in a secure environment (ie, privacy protection that is under the control of the Trial/Study PI), so that this resource could support the conduct of clinical trials. This approach is intended to ensure local governance control of the site PI, who can address the specific data protection conditions at diverse international locations.

In addition to prospective accumulation of individual new screening cases, existing imaging collections will remain at the local site where they were collected, so, again, the resulting environment remains consistent with local regulations without a privacy or data disclosure risk. An inherent design feature of ELIC is to manage large numbers of thoracic CT image screening registries. Therefore, control of the processes for the users of the spokes and hub requires a well-functioning informatics environment with easy-to-setup and deployment tools that will enable rapid screening care implementation as well as research by new global lung cancer screening groups.

The power of ELIC relates to the accessibility of the cloud and its remarkable cost efficiency.^16^ For image quality processes, ELIC already uses machine vision and will soon include artificial intelligence to ensure optimal and economic image quality. This resource can greatly accelerate radiomics and deep learning processes for medical images and can be integrated with digital pathology and genomic data. As the ELIC project continues to mature as a resource to conduct analyses and study international collections of high-quality thoracic CT images together with associated biomedical data, there are a number of models through which the pharmaceutical industry can be involved. For example, drug company sponsors may host their own ELIC spoke, on which they can collect quality-controlled CT images associated with new innovative neoadjuvant studies, adjuvant or even chemoprevention clinical trials, and associated clinical outcomes data. With each trial, the sponsor can decide which image collections to acquire and maintain as private versus aggregated in large public image collections. In this fashion, aggregated image and data collections could emerge as critical postmarketing research and quality resources. Furthermore, access to large quality-controlled CT screening images with clinical outcomes data will provide the basis on which to construct the next generation of algorithms to build models and tools, which can function as clinical decision support. This is a rapidly evolving area, and we are working to evolve ELIC in alignment with new regulatory guidance relative to responsibly building international clinical decision support tools for clinical care and research.^23-25^ Because thoracic CT images from screening also contain information about the presence of early coronary artery disease and chronic obstructive disease, a high-quality international collection of these images will be of growing public health value.^26^

In summary, the development and deployment of the ELIC hub-and-spoke environment, together with fair and internationally developed governance policies, will establish, to our knowledge for the first time, a large and efficient global computing environment for the study of thoracic CT scans obtained in the context of lung cancer screening. The use of QIBA CT Small Lung Nodule Profile and the associated phantoms and software tools will improve the quality of global thoracic CT images aggregated for ELIC, ensuring much more efficient image tool development. Not only will these resources help accelerate lung imaging research and the availability of thoroughly tested imaging tools, but the data generated will provide insights to guide future recommendations for lung screening and for managing early thoracic diseases.
